# Regional methylome profiling reveals dynamic epigenetic heterogeneity and convergent hypomethylation of stem cell quiescence-associated genes in breast cancer following neoadjuvant chemotherapy

**DOI:** 10.1186/s13578-019-0278-y

**Published:** 2019-02-07

**Authors:** Yumei Luo, Juan Huang, Yi Tang, Xitu Luo, Lingxia Ge, Xiujie Sheng, Xiaofang Sun, Yaoyong Chen, Detu Zhu

**Affiliations:** 1 Key Laboratory for Major Obstetric Diseases of Guangdong Province and Key Laboratory of Reproduction and Genetics of Guangdong Higher Education Institutes, The Third Affiliated Hospital of Guangzhou Medical University, Guangzhou, 510150 China; 2 Department of General Surgery, The Third Affiliated Hospital of Guangzhou Medical University, Guangzhou, 510150 China; 3 Department of Genetic Medicine, Weill Cornell Medical College, New York, NY 10065 USA

**Keywords:** Breast cancer, Neoadjuvant chemotherapy, Chemoresistance, DNA methylation, Epigenetic heterogeneity, High-density methylation microarray, Droplet digital PCR

## Abstract

**Background:**

Neoadjuvant chemotherapy (NAC) induces a pathological complete response (pCR) in ~ 30% of patients with breast cancer. However, aberrant DNA methylation alterations are frequent events during breast cancer progression and acquisition of chemoresistance. We aimed to characterize the inter- and intra-tumor methylation heterogeneity (MH) in breast cancer following NAC.

**Methods:**

DNA methylation profiles of spatially separated regions of breast tumors before and after NAC treatment were investigated using high-density methylation microarray. Methylation levels of genes of interest were further examined using multiplexed MethyLight droplet digital PCR (ddPCR).

**Results:**

We have discovered different levels of intra-tumor MH in breast cancer patients. Moreover, NAC dramatically altered the methylation profiles and such changes were highly heterogeneous between the patients. Despite the high inter-patient heterogeneity, we identified that stem cell quiescence-associated genes ALDH1L1, HOPX, WNT5A and SOX9 were convergently hypomethylated across all the samples after NAC treatment. Furthermore, by using MethyLight ddPCR, we verified that the methylation levels of these 4 genes were significantly lower in breast tumor samples after NAC than those before NAC.

**Conclusions:**

Our study has revealed that NAC dramatically alters epigenetic heterogeneity in breast cancer and induces convergent hypomethylation of stem cell quiescence-associated genes, ALDH1L1, HOPX, WNT5A and SOX9, which can potentially be developed as therapeutic targets or biomarkers for chemoresistance.

**Electronic supplementary material:**

The online version of this article (10.1186/s13578-019-0278-y) contains supplementary material, which is available to authorized users.

## Background

DNA methylation is a key mechanism for transcriptional regulation and is the best-studied epigenetic modification. Dramatic methylation changes of gene regulatory regions are associated with gene silencing or expression in promoters and enhancers. Recent large-scale genomic studies have shown that perturbations of methylation patterning are frequent events during breast cancer (BRCA) progression, and these aberrantly methylated genes are involved in cell cycle regulation, DNA repair, transformation, detoxification, adhesion and metastasis, such as BRCA1, CDH1, MGMT etc. [1]. Also, lots of published studies confirmed the important roles of DNA methylation modifications in patients’ resistance to standard chemotherapy treatments of BRCA. For example, hypermethylation of BRCA1 could predict the sensitivity to PARP inhibitors and alkylating agents [2, 3]; hypermethylation of GSTP1, ABCB1 and DUSP4 could predict the sensitivity to doxorubicin [4–6]; and hypermethylation of ESR1, CDK10 and PITX2 could predict the resistance to estrogen inhibitors [7, 8]. It is increasingly recognized that such epimutations can provide more insights to stratify subpopulations of tumor cells and evaluate treatment response when there are no well-known genetic mutations in specific tumors. Moreover, it is intriguing to consider epimutations as potential targets since it is less stable compared to genetic mutations and easier to be modified. Hence, DNA methylation profiling draws great interests to be used to evaluate chemotherapy response of BRCA in clinics.

Current availability of high-density DNA methylation microarray technology has enabled us to profile genome-wide DNA methylation signatures associated with drug response more efficiently. Moreover, the high density of probes also enables us to examine copy number variation (CNV) in the tumor tissue with comparable sensitivity of SNP arrays [9, 10]. Furthermore, it is reported that the arrays are sufficiently sensitive to evaluate the abundance of tumor-infiltrated lymphocytes (TILs) in BRCA tumors based on certain methylation signature, which reflects the strength of antitumor immune response and can serve as a potential biomarker of survival and response of chemotherapy [11].

However, intra-tumor heterogeneity may lead to sampling bias and pose a major challenge to biomarker development and precision medicine [12]. Generally, there are two levels of methylation heterogeneity (MH), including inter- and intra-tumor MH. People often pay more attention to the former through collecting more and more samples from different backgrounds [13]. However, there are growing evidences showing that intra-tumor MH is more dominant for evaluating the response upon treatment for an individual patient [14]. Despite many studies characterizing intra-tumor genetic heterogeneity, epigenetic heterogeneity of DNA methylation has been less investigated in BRCA.

In this study, we profiled MH of spatially separated regions of breast tumors prior to and post neoadjuvant chemotherapy (NAC) treatment using the high-density Infinium HumanMethylation450K bead arrays. Overall, we have discovered different levels of intra-tumor MH in BRCA patients. Moreover, NAC dramatically altered the methylation profiles and changes were heterogeneous between different individuals. Despite the high inter- and intra-tumor heterogeneity, we identified that stem cell quiescence-associated genes ALDH1L1, HOPX, WNT5A and SOX9 were commonly hypomethylated across all the samples post NAC treatment, which holds the potential to be developed as therapeutic targets or biomarkers for chemoresistance.

## Methods

### Patient materials

This study had obtained the approval of the Ethics Committee of the Third Affiliated Hospital of Guangzhou Medical University (2017/056). Patients were enrolled at the Third Affiliated Hospital of Guangzhou Medical University (Guangzhou, China) and had signed informed consents. 3 BRCA patients (Patients 602, 676 and 164) who received NAC treatments but failed to achieve pathologic complete response (pCR) were selected. These patients underwent core needle biopsy sampling, followed by Cyclophosphamide, Epirubicin and Docetaxel neoadjuvant chemotherapy regimens, and finally surgical removal of the breast tumors. Each patient derived 3–4 core needle biopsy specimens prior to NAC, and each post-NAC tumor tissue was spatially dissected into 6–7 sectors (Additional file 1: Figure S5A). Another 5 breast cancer patients (Patients 161, 486, 168, 533 and 847) were selected whose breast tumors were surgically removed without NAC treatment. Each tumor tissue was spatially dissected into six sectors (Additional file 1: Figure S5B). DNA of the samples was isolated using the DNeasy Blood & Tissue Kit (Qiagen) and then bisulfite converted using the EZ DNA Methylation Kit (ZymoResearch) according to the manufacturers’ instructions.

### DNA methylation profiling

Genome-wide methylation profiling of 47 breast cancer tissue samples derived from the 8 patients was performed using the Illumina Infinium HumanMethylation450k Bead Chip according to the manufacturer’s instruction. IDAT raw data files were imported for processing using the R/Bioconductor package methylumi pipeline with the default parameters [15]. The output of methylumi pipeline contains beta values (methylation signal density/total signal density) with annotations for the HUGO Gene Nomenclature Committee (HGNC) gene symbol, chromosome, and genomic coordinate of each CpG/CpH site (UCSC hg19). Methylation levels (beta values) were used for the subsequent analyses.

### Copy number alteration analysis

ChAMP [16], an R package available through Bioconductor, was used for evaluating copy number variation (CNV) with default options, by comparing each sample with internal 450k blood control samples. Segmentation was performed by champ. CNA function, which utilized the intensity values from HumanMethylation450 BeadChip probes to count copy number and determined if copy number alterations were present. Copy number was determined using the CopyNumber package. The log2-ratio value for each segment was calculated by using the sum of the methylated and unmethylated signal density. The CNVs for each sample were plotted according to the log2-ratio from each segment, and classified as amplifications (> 0.08) or deletions (< − 0.08).

### Differential methylation analysis and functional enrichment analysis

DMRcate [17], an R package available through Bioconductor, was employed for detecting differentially methylated regions (DMR) between samples before and after NAC for each patient individually. DMRs were defined as regions with a maximal 1000 bp containing two or more CpGs. FDR were calculated with Benjamini–Hochberg procedure, and FDR cutoff 0.05 was used to determine DMRs. Genomic locations for all DMRs were assigned by DMRcate to Illumina hg19 annotation. Genes whose promoter region overlap with DMRs in all samples were selected as common DM genes for the downstream analysis. Functional analysis was performed on DM genes with DAVID (version 6.8) [18] and KEGG pathways with enrichment P < 0.05 were selected as overrepresented functions.

### MethyLight droplet digital PCR

MethyLight droplet digital PCR (ddPCR) was performed to validate the methylation levels of the genes of interest in tumor biopsies derived from additional NAC-treated breast cancer patients. The ddPCR reaction mixture consisted of the bisulfite-converted DNA sample, ddPCR Supermix for Probes (BioRad), and locus-specific MethyLight primers and probes in a final volume of 20 µL. The primer and probe sequences were designed using Beacon Designer version 8.20 (Premier Biosoft). The sequences of the primers and probes are listed in Additional file 1: Table S2. The locations of the primers and probes are shown in Additional file 1: Figure S11A. A multiplexed MethyLight ddPCR assay, as described in Additional file 1: Figure S11B, was established to simultaneously quantified 2 genes of interest (2 gene-specific FAM-labelled probes adjusted at different concentrations) and the C-LESS-C1 reaction (HEX-labelled probe), which amplified a DNA strand without any cytosine to determine the total DNA amounts of each sample [19]. This multiplexed method enabled us to quantify the 4 genes with only 2 reactions. The ddPCR reaction mixtures were loaded into sample wells on a DG8 Cartridge (BioRad). A volume of 70 µL of droplet generation oil was loaded into adjacent oil wells on the cartridge. Then the cartridge was loaded into a QX200 Droplet Generator (BioRad) for droplet generation. The resulting water-in-oil droplets were gently transferred from the droplet wells on the cartridge to a 96-well PCR plate (BioRad). The plate was heat-sealed with PX1TM PCR Plate Sealer (BioRad), placed on a T100TM Thermal Cycler (BioRad) and amplified to the endpoint. After PCR amplification, the plate was loaded into a QX200 Droplet Reader (Bio-Rad) to determine how many droplets were positive for the genes of interest, as well as for the control reaction C-LESS-C1. Data were analyzed using QuantaSoft version 1.4.0 (BioRad). The methylation levels of the genes of interest in each sample were normalized by the total DNA amounts based on the C-LESS-C1 reaction.

## Results

### DNA methylation provides independent information from current BRCA classification systems

It is well known that BRCA has clinical and genomic heterogeneity. Traditionally, BRCA has been staged by histopathological criteria that are based on size, lymph node infiltration and level of invasiveness (TNM), or by immunohistochemical characterization of cell surface receptors, including estrogen receptor (ER), progesterone receptor (PR) and human epidermal growth factor receptor 2 (HER2) [20]. Recently, gene expression profiles are employed to expand the molecular classification of BRCA. One of the most well-known signatures is PAM50, which is used to distinguish 5 intrinsic subtypes, including luminal A, luminal B, HER2-enriched, basal-like and normal-like [21]. Considering that DNA methylation is one of the critical regulators of gene expression, we seek to study if subtypes of BRCA based on current classifications display consistent DNA methylation changes.

To fulfill this task, we downloaded 450K array data of 870 patients from the TCGA-BRCA study and performed unsupervised clustering analysis with the most variable probes (top 1%). Generally, we observed great inter-patient MH across the whole cohort and no significant correlation between certain methylation patterns and PAM50 subtypes, surface receptors or TNM stages was found (Additional file 1: Figures S1–S3). Thus, all current classification methods, no matter based on gene expression profiles, surface receptors or histopathological criteria, were not significantly correlated with DNA methylation landscapes. Therefore, it is intriguing to utilize DNA methylation to expand the prediction power to clinical outcomes of NAC treatment since DNA methylation can provide independent information from traditional classifiers.

### Primary BRCA tumors display different levels of intra-tumor MH

To investigate the intra-tumor MH in BRCA patients and the impact of NAC on it, we have selected 3 BRCA patients (Patients 602, 676 and 164) who received NAC treatment but failed to achieve pCR (Additional file 1: Figure S4, Table S1). Each patient derived 3–4 pre-NAC biopsies and the post-NAC surgically-excised tumors were spatially dissected into 6–7 sectors (Additional file 1: Figure S5A). We also included another 5 patients (Patients 161, 486, 168, 533 and 847) with their tumor surgically excised without any NAC treatment (Additional file 1: Figure S4, Table S1). Each tumor tissue was spatially dissected into 6 sectors (Additional file 1: Figure S5B). Samples with successful DNA exaction were used for DNA methylation array assays.

**Fig. 1:**
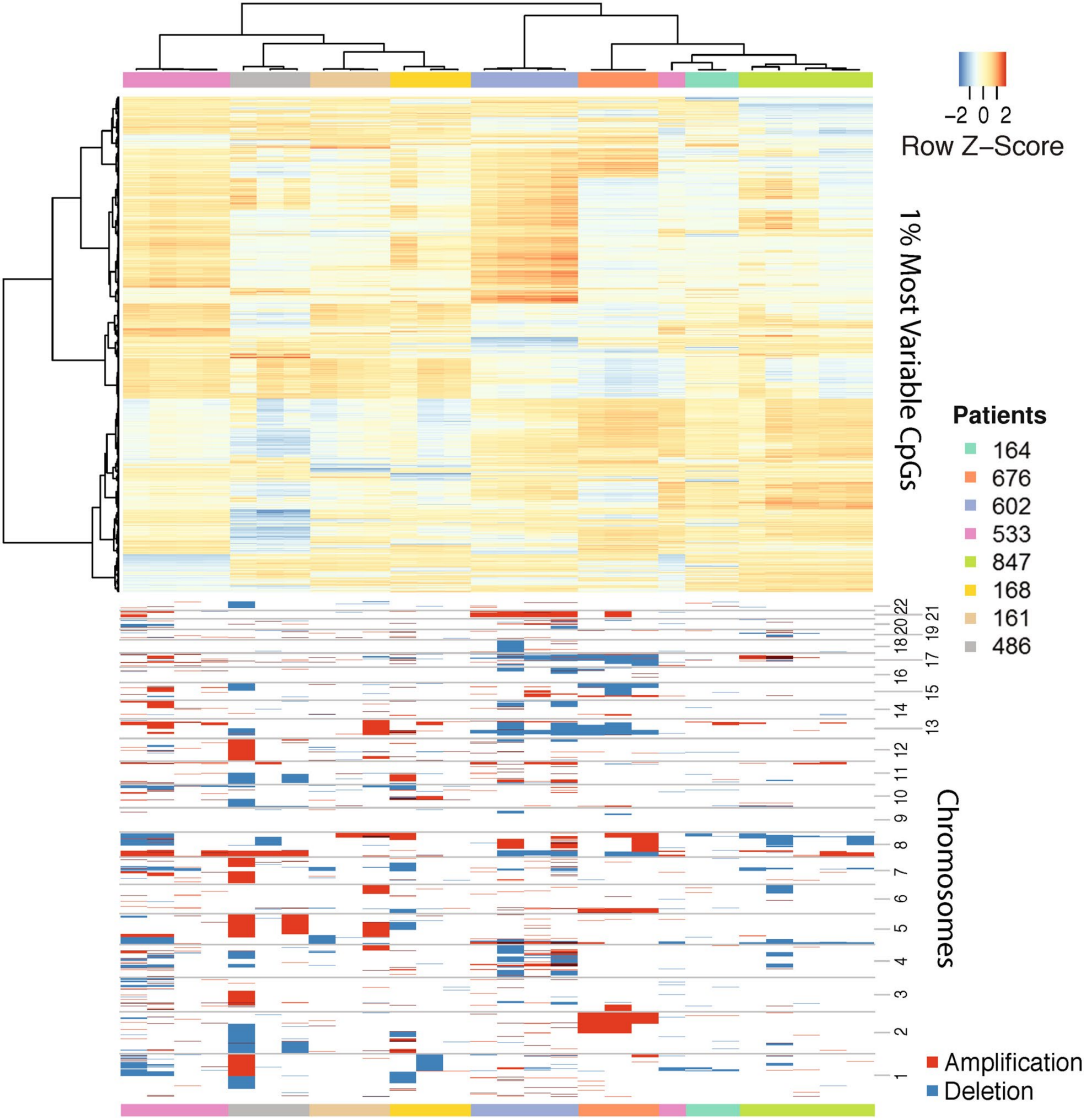
Differences of genetic and epigenetic profiles between baseline samples from different patients. Top panel: unsupervised clustering of top 1% most variable probes of 450K data from baseline samples prior to NAC; Bottom panel: copy number plots of corresponding samples

Firstly, we characterized the DNA methylation profiles of pre-NAC samples with top 1% most variable CpGs cross all the samples. As expected, we observed high levels of inter-patient MH, which was consistent with our conclusions from the TCGA cohort (Fig. 1, top panel). By comparing different samples in each patient individually, we observed different levels of intra-tumor MH. Some patients had relatively lower levels of intra-tumor MH, such as Patients 161, 602, 676 and 164. All the samples from the same patient shared very similar methylation profiles. Some patients had relatively higher levels of intra-tumor MH, such as Patients 486, 168 and 847. In these patients, samples from the same patient show different methylation profiles in partial CpGs. However, these samples were still clustered together by individuals, which indicated such intra-tumor MH level was lower than inter-patient MH. For patient 533, one of the samples was quite different from the others. These results indicated that primary BRCA patients had different levels of intra-tumor MH. Besides the overall analysis based on all samples, we also performed the unsupervised clustering on samples from each patient individually. The top 1% most variable CpGs from each patient showed very different profiles, which indicated that each patient carried distinct MH patterns (Additional file 1: Figure S6).

Leveraging the power of high-density probes of 450K array, we also evaluated the genetic heterogeneity of CNV at the same time. Similar with methylation profiles, we observed high levels of inter-patient genetic heterogeneity, and different patients had different levels of CNVs and different focal CNVs in almost every chromosome (Fig. 1, bottom panel). However, we found the levels of intra-tumor genetic heterogeneity were not correlated to those of intra-tumor MH. For example, Patient 161 had almost homogeneous methylation profiles but quite diverse CNV landscapes, especially on chromosomes 5, 6 and 13; Patient 847 had high levels of both genetic and epigenetic intra-tumor heterogeneities.

To further examine the effects of intra-tumor MH on detecting known methylation-based biomarkers, we performed unsupervised clustering analysis on the 450K array probes mapped to 7 reported chemotherapy-resistant genes that were epigenetically regulated, including DUSP4, GSTP1, ABCB1, PTEN, FOXC1, TGM2 and ETS1 [4–6, 22]. We observed that most samples were clustered by individuals. However, samples from Patients 486, 847 and 533, who had relatively higher intra-tumor MH, were fallen into two different clusters (Additional file 1: Figure S7). We also tested another 5-probe methylation signature of TIL (MeTIL) that was reported to reflect TIL abundance and predict chemotherapy outcomes [11]. Similarly, we observed that the samples from Patients 168, 847 and 533, whose intra-tumor MHs were relatively higher, were even separated into three different clusters (Additional file 1: Figure S8). These observations indicated that higher intra-tumor MH might cause higher inter-sample variance of the methylation markers, thus leading to sampling bias. Therefore, intra-tumor MH needs to be taken into consideration when we stratify BRCA patients and perform personalized medicine.

### BRCA tumors exhibit heterogeneous DNA methylation changes in response to NAC

**Fig. 2:**
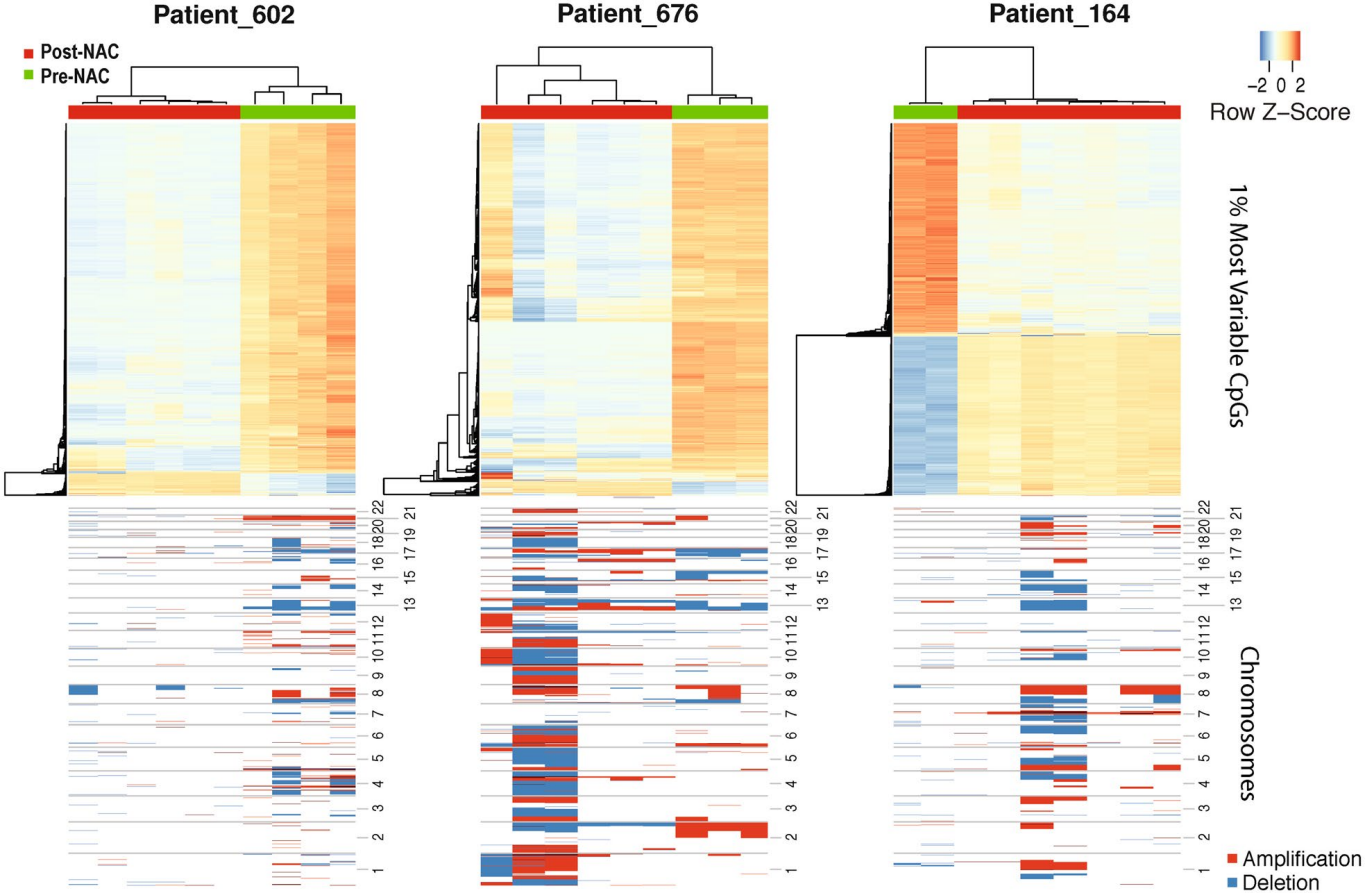
Differences of genetic and epigenetic profiles between baseline and chemo samples from three different patients. Top panel: unsupervised clustering of top 1% most variable probes of 450K data for each patient who participated chemotherapy separately; Bottom panel: copy number plots of corresponding samples

To investigate how NAC changes DNA methylation profiles in BRCA patients, we applied unsupervised clustering analysis to the 3 NAC-treated patients. As the result, we observed that NAC treatment changed methylation profiles dramatically in all the 3 patients (Fig. 2, top panel). Surprisingly, the top 1% variable CpGs were altered very differently across the patients, indicating that NAC changed methylation profiles in divergent ways. In Patients 602 and 676, the majority of top variable CpGs were hypo-methylated after NAC. However, in Patient 164, the methylation changes were almost balanced, with nearly half of CpGs hyper-methylated and the others hypo-methylated after NAC. Taken together, these results provided the evidence that NAC could significantly alter the overall methylation profiles and cause heterogeneous methylation changes in different BRCA patients.

In terms of CNVs, we also observed heterogeneous changes induced by NAC in different patients (Fig. 2, bottom panel). In Patients 244676 and 248164, post-NAC samples gained more CNVs compared to pre-NAC samples. In Patient 602, the opposite CNV change was observed. Even more interestingly, in Patients 676 and 164, the post-NAC samples actually gained CNVs to different extends, indicating increased intra-tumor genetic heterogeneity caused by NAC treatment. All these observations proposed that BRCA patients would exhibit heterogeneous responses to NAC treatment at both genetic and epigenetic levels, and DNA methylation changes were independent with CNV changes.

### Heterogeneous epigenetic regulations on cancer-related pathways by NAC

**Fig. 3:**
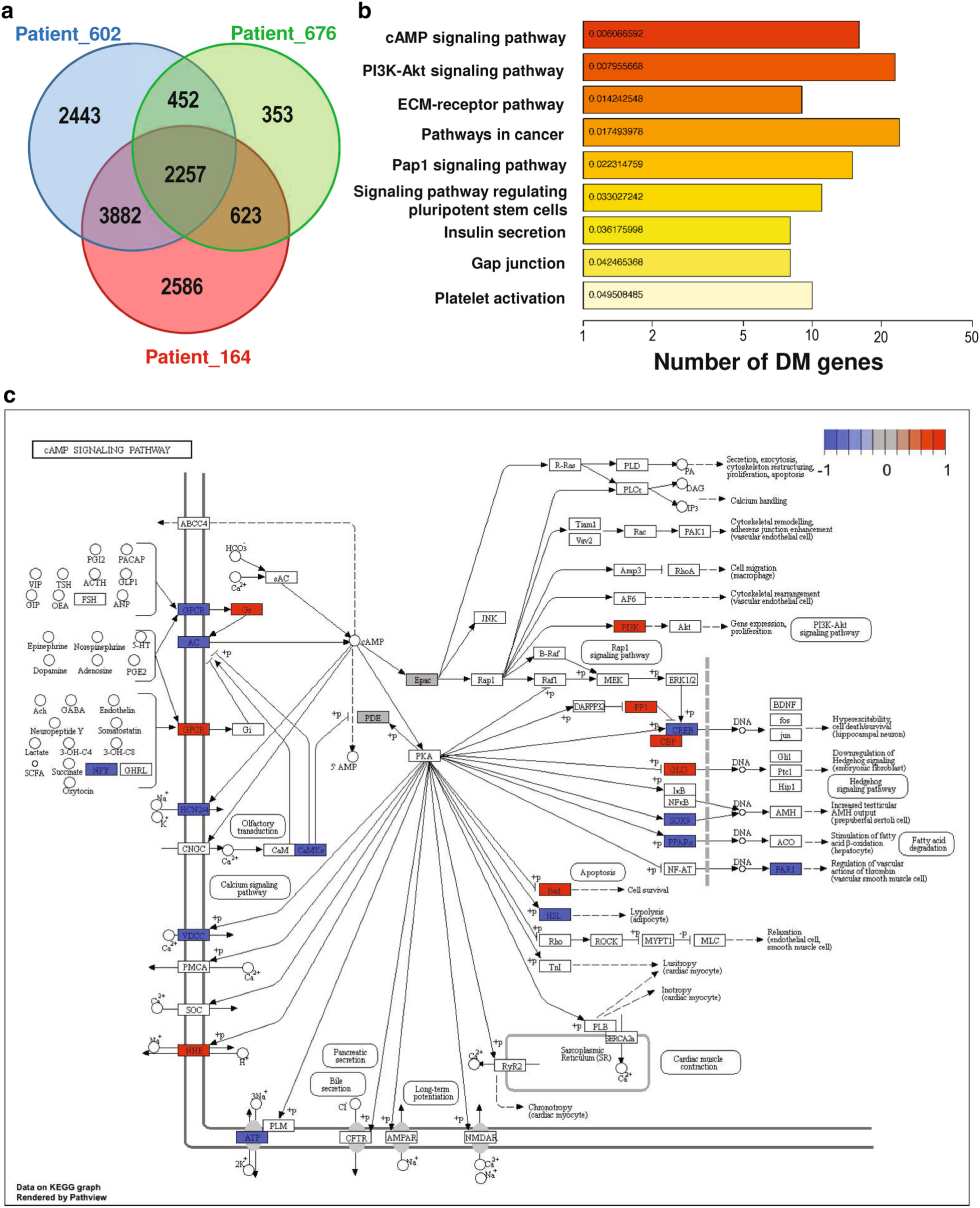
Differential methylation analysis of the 3 NAC-treated patients. **a** Venn diagram of DM gene numbers of each patient; **b** top enriched KEGG pathways based on common DM genes; **c** common DM genes in the cAMP signaling pathway

To investigate the genes involved in NAC-induced DNA methylation alterations, we performed supervised analysis on the DNA methylation levels of CpGs and defined DMRs between samples prior to and post NAC for each patient. Thus, we detected 9348, 3685 and 9034 DMRs for Patients 164, 676 and 602, respectively (Fig. 3a). Finally, 2257 common DMRs were found among all the 3 patients and these DMRs were assigned to the nearest genes with hg19 annotation. This indicated that NAC could profoundly lead to the robust epigenetic changes on a group of genes regardless of patients’ genomic and epigenetic variances. To better understand the underlying mechanism of these regulations, pathway enrichment analysis was performed on above differentially methylated (DM) genes. Interestingly, as the most prominent functional categories of DM genes, a group of cancer-related pathways were significantly altered by NAC, including cAMP signaling pathway, PI3K–AKT signaling pathway, ECM-receptor interaction etc. (Fig. 3b). A closer look at the cAMP signaling pathway showed that although many genes in the pathway were differentially methylated across all the 3 patients (Fig. 3c), most of them were altered in divergent ways (Additional file 1: Figure S9). For instance, in Patient 676, most of the DM genes were hypomethylated; while the other 2 patients displayed the opposite. A further look at the other cancer-related pathways in each patient showed that different pathways were regulated in divergent ways as well, which implied that breast tumors in different patients might gain NAC resistance via different molecular mechanisms (Additional file 1: Figure S10).

### Four stem cell quiescence-associated genes were convergently hypomethylated by NAC

**Fig. 4:**
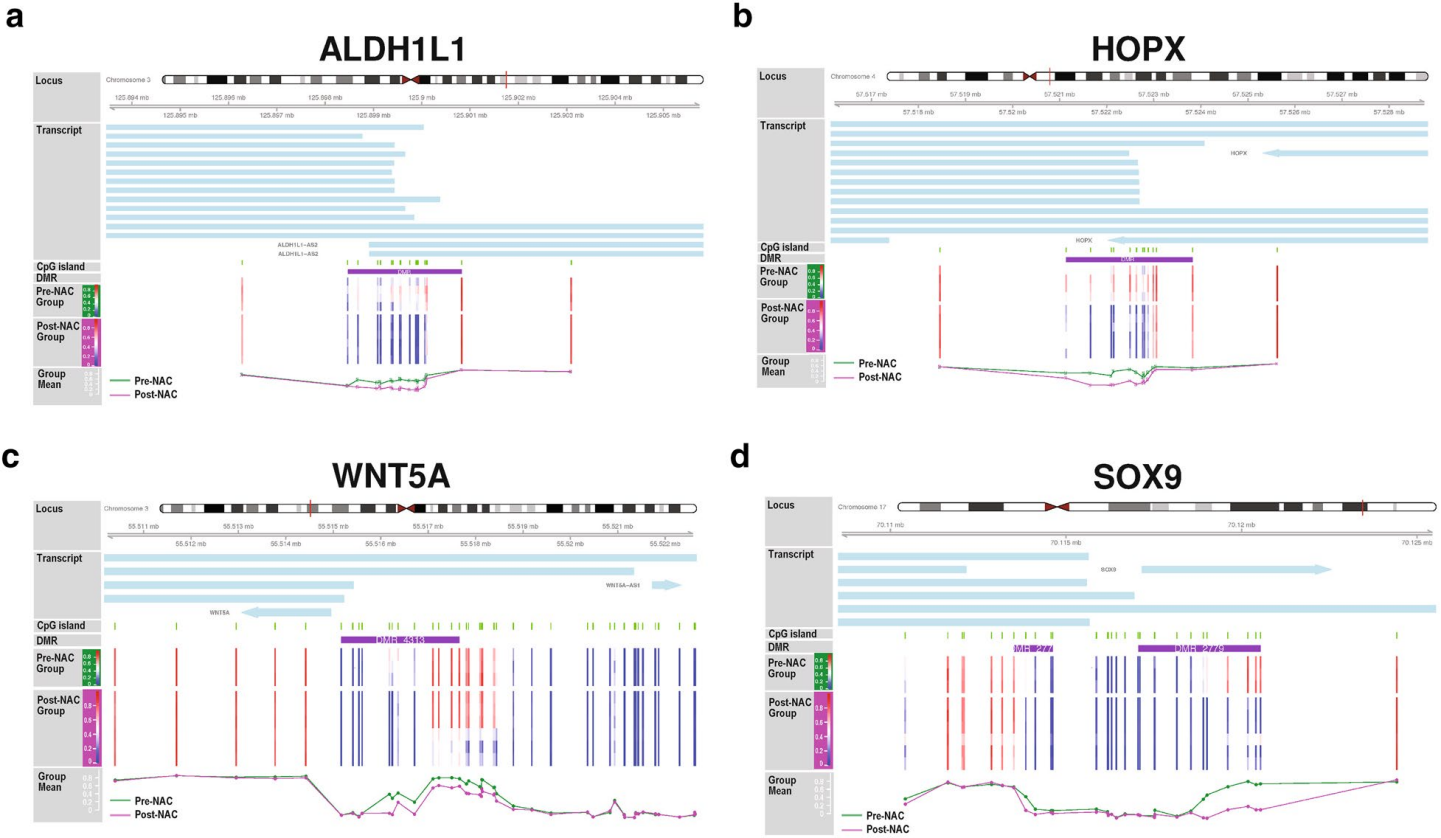
Comparison of methylation levels between pre- and post-NAC samples in the promoter regions of the top DM genes **a** ALDH1L1; **b** HOPX; **c** WNT5A; **d** SOX9

Despite the high inter- and intra-tumor heterogeneity, we hypothesize that there might be a small but common methylation signature for NAC resistance in which selective pressure by chemotherapy converges at specific genes. We looked into the top DM genes and found that 4 genes, ALDH1L1, HOPX, WNT5A and SOX9, were convergently hypomethylated across all the post-NAC samples (Fig. 4). From the literature, the functions of these 4 genes are associated with stem cell quiescence [23–27].

**Fig. 5:**
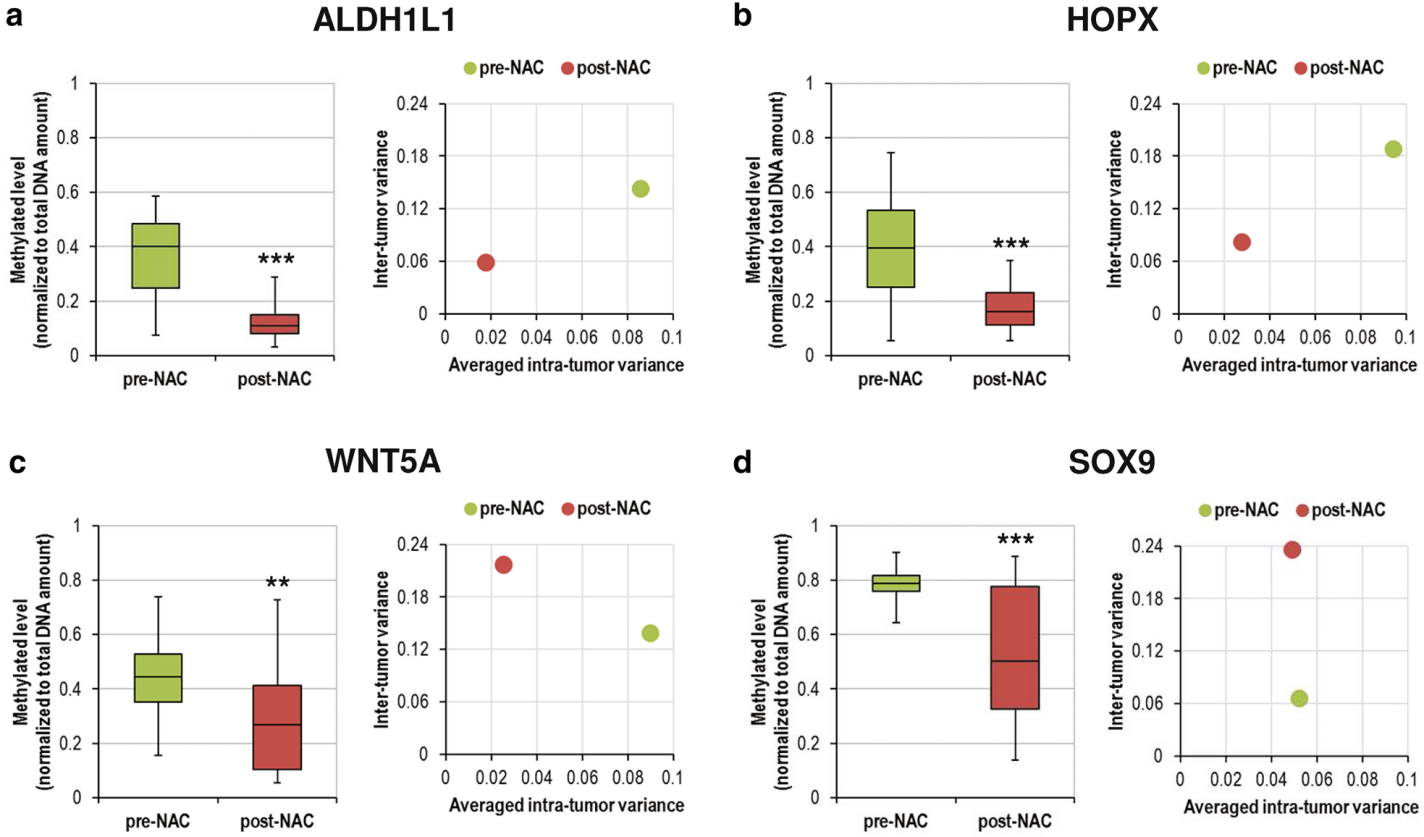
MethyLight ddPCR analysis of methylation levels of the genes of interest in pre- and post-NAC tumor samples. The boxplots show the pre- and post-NAC methylation levels in the 24 tumor samples. The scatter plots show the inter-tumor variance and averaged intra-tumor variance of the pre- and post-NAC groups. **a** ALDH1L1; **b** HOPX; **c** WNT5A; **d** SOX9. **P < 0.01, ***P < 0.001

We further examined the methylation status of these 4 genes in additional 24 NAC-treated BRCA using multiplexed MethyLight droplet digital PCR (ddPCR) assays (Additional file 1: Figure S11). At least 3 pieces of spatially separated pre- and post-NAC tumor biopsies were collected from each patient. The results confirmed that these 4 genes are significantly less methylated in post-NAC tumor samples (Fig. 5). We also looked into the alteration in intra-tumor MH of these 4 genes under NAC pressure. All genes except SOX9 displayed decreased intra-tumor variance of methylation levels after NAC treatment, indicating most of them were subjected to convergent selection pressure by NAC (Fig. 5). The pre-NAC intra-tumor variance of SOX9 was already much lower than those of the other 3 genes, so it was reasonable that the post-NAC variance did not lower more (Fig. 5). Further, we looked into the alteration in inter-tumor MH of these 4 genes under NAC pressure. Among the 4 genes, ALDH1L1 and HOPX exhibited decreased inter-tumor variance of methylation levels after NAC treatment; meanwhile, WNT5A and SOX9 showed increased inter-tumor variance (Fig. 5). This indicated that there might be higher selection pressure on ALDH1L1 and HOPX by NAC treatment. In summary, the convergent hypomethylation of these 4 genes in tumor biopsies after NAC was validated by MethyLight ddPCR assays.

## Discussion

Although as one of most well-studied cancer types, BRCA has been classified to distinct subtypes according to the different criteria for determining the treatment strategy [20], it is still not robust to predict prognosis or therapeutic response with either pathological or immunochemical characterization due to the heterogeneity of the disease. Recent large-scale genomic studies have shown that mutations in the epigenetic machinery and concomitant perturbation of epigenomic patterning are frequent events in BRCA [28]. The best-studied epigenetic modification is DNA methylation, hyper- or hypo-methylation of gene regulatory regions is associated with gene silence or activation. For example, methylation of the p16 tumor suppressor gene was a potential early biomarker for detection of BRCA [29]. Tumor DNA methylation has two levels of heterogeneity, one is inter-patient MH and the other one is intra-tumor MH. Currently, lots of studies were focusing on inter-tumor MH, and lots of data were generated by large-scale projects, such as TCGA. However, studies focusing on intra-tumor MH patients are very limited so far due to limitations of technologies and cost. It has been reported that intra-tumor MH is predictive to tumor progression in many types of tumors especially in lymphomas [30]. Nevertheless, the intra-tumor MH in BRCA and its potential impact on breast cancer progression and acquisition of drug resistance are less studied. Our study firstly addresses the intra-tumor MH in primary breast tumors in a genome-wide manner and in the context of NAC.

Our results indicated that primary BRCA tumors had great inter-tumor MH and different levels of intra-tumor MH. Hence, if we want to use methylation-based biomarkers to predict clinical outcomes, intra-tumor MH is one of the most important features to be considered. For heterogeneous patients, we need to evaluate different regions of the tumors and make predictions taking such variable information into consideration.

Moreover, we observed that the patients exhibited heterogeneous DNA methylation and CNV changes in response to NAC, suggesting that even the same regime might affect different patients in divergent ways at both genetic and epigenetic levels.

Furthermore, we observed heterogeneous epigenetic regulation on cancer-related pathways by NAC, including cAMP signaling pathway, PI3K–AKT signaling pathway, ECM-receptor interaction etc. It is reported that the cAMP signaling pathway has both pro- and anti-apoptotic roles in cancers [31]. Some studies showed that anti-cancer drugs such as cisplatin, ABT-737 and thymoquinone induced cancer cell apoptosis via the cAMP/PKA axis [32, 33]. Some other studies demonstrated that hyperactivation of the cAMP/PKA axis conferred multidrug resistance in ovarian cancer [34, 35]. Considering the other cancer-related pathways were also heterogeneously regulated in our study, we hypothesize that the cAMP signaling pathway functions in BRCA in a context-dependent manner. Hence, our results indicated that it is important to gain genome-wide information when we perform precision medicine for BRCA patients.

In spite of the high inter-patient heterogeneity, we identified that 4 stem cell quiescence-associated genes, ALDH1L1, HOPX, WNT5A and SOX9, were convergently hypomethylated in all the post-NAC samples. ALDH1L1 is a key enzyme that negatively regulate the one-carbon metabolism to limit cell proliferation [36, 37] and is a marker for quiescent neuron stem cells [23]. WNT5A is a non-canonical Wnt ligand and functions to maintain the quiescent state of multiple adult stem cells, such as hematopoietic stem cells [24] and neural stem cells [25]. HOPX is a transcription factor that negatively regulates cell proliferation and is expressed in multiple quiescent adult stem and progenitor cells, such as intestinal stem cells, hair follicle stem cells and cardiac progenitors [26]. Similar with HOPX, SOX9 is transcription factor that regulates quiescence of intestinal stem cells and hair follicle stem cells [27]. More interestingly, it is reported to reprogram tumor cell into dormant status as well [38]. As NAC is more effective for rapidly proliferating tumor cells, we hypothesize that the hypomethylation of these quiescence-associated genes might enable the tumor cells to escape NAC by driving them into dormancy. Novel synthetic biology tools, such as the CRISPR system [39, 40] and the combinatorial transgene expression system [41, 42], can be used to establish relevant cellular models to further investigate the underlying mechanisms.

## Conclusion

In conclusion, our findings have demonstrated that NAC dramatically alter both inter- and intra-tumor MH in BRCA tumors. Furthermore, 4 stem cell quiescence-associated genes ALDH1L1, HOPX, WNT5A and SOX9 are convergently hypomethylated in BRCA tumors after NAC treatment, which hold the potential to developed as therapeutic targets or biomarkers for chemoresistance.

## Additional file

**Additional file 1: Table S1.** Clinical Characteristics of Selected Breast Cancer Patients. **Table S2.** Primers and probes used in ddPCR MethyLight assays. **Figure S1.** Unsupervised clustering of top 1% most variable probes of all the 870 TCGA-BRCA 450K samples. **Figure S2.** Unsupervised clustering of top 1% most variable probes of all the 870 TCGA-BRCA 450K samples with PR/ER/HER2 annotations. **Figure S3.** Unsupervised clustering of top 1% most variable probes of all the 870 TCGA-BRCA 450K samples with TNM annotations. **Figure S4.** Histopathological analysis of selected breast cancer patients. **Figure S5.** Sampling procedures. **(A)** Breast cancer patients were selected who underwent core needle biopsy sampling, followed by CET neoadjuvant chemotherapy regimens, and finally surgical removal of the breast tumors. Each patient derived 3-4 core needle biopsy specimens prior to NAC, and each post-NAC tumor tissue was spatially dissected into 6-7 sectors; **(B)** Breast cancer patients were selected whose breast tumors were surgically removed without neoadjuvant chemotherapy treatment. Each tumor tissue was spatially dissected into 6 sectors. **Figure S6.** Unsupervised clustering of top 1% most variable probes of samples from 5 patients without chemotherapy separately. **Figure S7.** Unsupervised clustering of 450K array probes mapped to known genes associated to chemotherapy resistant: ABCB1, DUSP4, ETS1, FOXC1, GSTP1, PTEN and TGM2. **Figure S8.** Unsupervised clustering of MeTIL signature probes of all the samples. **Figure S9.** DM genes in KEGG cAMP signaling pathway in the 3 NAC-treated patients. Red color indicates hypermethylated genes; blue color indicates hypomethylated genes. **Figure S10.** DM genes in KEGG Pathways in Cancer in the 3 NAC-treated patients. Red color indicates hypermethylated genes; blue color indicates hypomethylated genes. **Figure S11.** Establishment of multiplexed Methylight ddPCR. **(A)** Locations of CpGs in the MethyLight primers and probes and the amplicons for methylated loci of interest, and the C-LESS-C1 assay that amplifies a DNA strand without any cytosine to determine the total DNA amounts in each sample. Genomic coordinate is referred to UCSC hg19; **(B)** The C- LESS-C1 assay is measured by the HEX-labelled probe, meanwhile 2 genes of interest are measured by the 2 genespecific FAM-labelled probes adjusted at different concentrations. The accuracy of ddPCR is sufficient to display the 2 gene-specific FAM-positive droplets at 2 distinctively separated FAM altitudes, enabling quantification of the 2 genes with 1 fluorescent channel. For the first assay, Gene A = ALDH1L1, Gene B = SOX9; for the second assay, Gene A = HOPX, Gene B = WNT5A. Thus, the 4 genes can be measured with only 2 assays.

## Abbreviations

BRCA: breast cancer
CNV: copy number variation
ddPCR: digital droplet polymerase chain reaction
DMR: differentially methylated region
MH: methylation heterogenetiy
NAC: neoadjuvant chemotherapy
TIL: tumor-infiltrated lymphocyte
