# Supplementary material for: Regional methylome profiling reveals dynamic epigenetic heterogeneity and convergent hypomethylation of stem cell quiescence-associated genes in breast cancer following neoadjuvant chemotherapy
Source: Cell Biosci. 2019 Feb 7;9:16. doi: 10.1186/s13578-019-0278-y (PMC6367786; doi:10.1186/s13578-019-0278-y)
Supplement: Supplementary file 1 — Additional file 1: Table S1. Clinical Characteristics of Selected Breast Cancer Patients. Table S2. Primers and probes used in ddPCR MethyLight assays. Figure S1. Unsupervised clustering of top 1% most variable probes of all the 870 TCGA-BRCA 450K samples. Figure S2. Unsupervised clustering of top 1% most variable probes of all the 870 TCGA-BRCA 450K samples with PR/ER/HER2 annotations. Figure S3. Unsupervised clustering of top 1% most variable probes of all the 870 TCGA-BRCA 450K samples with TNM annotations. Figure S4. Histopathological analysis of selected breast cancer patients. Figure S5. Sampling procedures. (A) Breast cancer patients were selected who underwent core needle biopsy sampling, followed by CET neoadjuvant chemotherapy regimens, and finally surgical removal of the breast tumors. Each patient derived 3-4 core needle biopsy specimens prior to NAC, and each post-NAC tumor tissue was spatially dissected into 6-7 sectors; (B) Breast cancer patients were selected whose breast tumors were surgically removed without neoadjuvant chemotherapy treatment. Each tumor tissue was spatially dissected into 6 sectors. Figure S6. Unsupervised clustering of top 1% most variable probes of samples from 5 patients without chemotherapy separately. Figure S7. Unsupervised clustering of 450K array probes mapped to known genes associated to chemotherapy resistant: ABCB1, DUSP4, ETS1, FOXC1, GSTP1, PTEN and TGM2. Figure S8. Unsupervised clustering of MeTIL signature probes of all the samples. Figure S9. DM genes in KEGG cAMP signaling pathway in the 3 NAC-treated patients. Red color indicates hypermethylated genes; blue color indicates hypomethylated genes. Figure S10. DM genes in KEGG Pathways in Cancer in the 3 NAC-treated patients. Red color indicates hypermethylated genes; blue color indicates hypomethylated genes. Figure S11. Establishment of multiplexed Methylight ddPCR. (A) Locations of CpGs in the MethyLight primers and probes and the amplicons for methylated loci of [file 13578_2019_278_MOESM1_ESM.pdf]

Table S1. Clinical Characteristics of Selected Breast Cancer Patients

Patient ID	Age	TNM stage	Clinical stage	Histological grade	Immunohistochemical characteristics								Tumor size	Neoadjuvant chemotherapy			
					HER2	ERα	PR	CK5/6	EGFR	E-cadhrin	P53	Ki-67		Cyclophosphamide	Epirubicin	Docetaxel	Cycle
164	47	T2N1M0	2B	III	+	+	-	+++	+	+++	+	60%	2.4cm	0.9g	110mg	120mg	3
676	41	T3N0M0	2B	III	-	+	-	+	+++	+++	+++	80%	8cm	0.88g	130mg	130mg	4
602	50	T3N1M0	3A	III	+++	+	-	++	+	+++	+++	30%	6.0cm	0.8g	110mg	120mg	4
533	53	T2N1M0	2B	III	+++	++	++	++	++	+++	-	40%	4.2cm	NA	NA	NA	NA
847	26	T1N0M0	1	III	-	++	++	+	+	+++	+++	60%	2.0cm	NA	NA	NA	NA
168	45	T2N1M0	2B	III	+++	+	-	+	++	+++	+++	80%	2.6cm	NA	NA	NA	NA
161	42	T2N1M0	2B	II	+	++	++	+	+	+++	+++	50%	2.5cm	NA	NA	NA	NA
486	89	T2N0M0	2A	III	+	+++	++	+++	+++	+++	+++	50%	4cm	NA	NA	NA	NA

Table S2. Primers and probes used in ddPCR MethyLight assays

**Assays**	**Probe/Primer**	**Sequences**
ALDH1L1	Probe	FAM-5' CGTTTATCGGTGGTGGGAT 3'-BHQ1
	Forward	5' CATTTATAAACCCGCCCGAAA 3'
	Reverse	5' AAATACGCTACGCTCCCGCCAT 3'
HOPX	Probe	FAM-5' ACCCGAATTCCACCACGCTATACCTCATC 3'-BHQ1
	Forward	5' CGGAAAGGTTTGTTTCGGTCG 3'
	Reverse	5' AAATCCTAAAATACAACTTCAACAAAATCG 3'
WNT5A	Probe	FAM-5' CCAACTACGACTCGAAAACCAACGCCT 3'-BHQ1
	Forward	5' TTGTAGATGAGTTTAGACGGGGTTA 3'
	Reverse	5' AACCTCTTTACCCTAAAATCGATACG 3'
SOX9	Probe	FAM-5' CGCTAAAACTAACCCGACTCGCTACTCAAC 3'-BHQ1
	Forward	5' GGTATTTTCGTAGTAGTTATAGGCGTA 3'
	Reverse	5' AACTCAACTACTCCGTCTTAATATACG 3'
C-LESS-C1	Probe	HEX-5' CCTCCCCCTCTAACTCTAT 3'-BHQ1
	Forward	5' TTGTATGTATGTGAGTGTGGGAGAGAGA 3'
	Reverse	5' TTTCTTCCACCCCTTCTCTTCC 3'

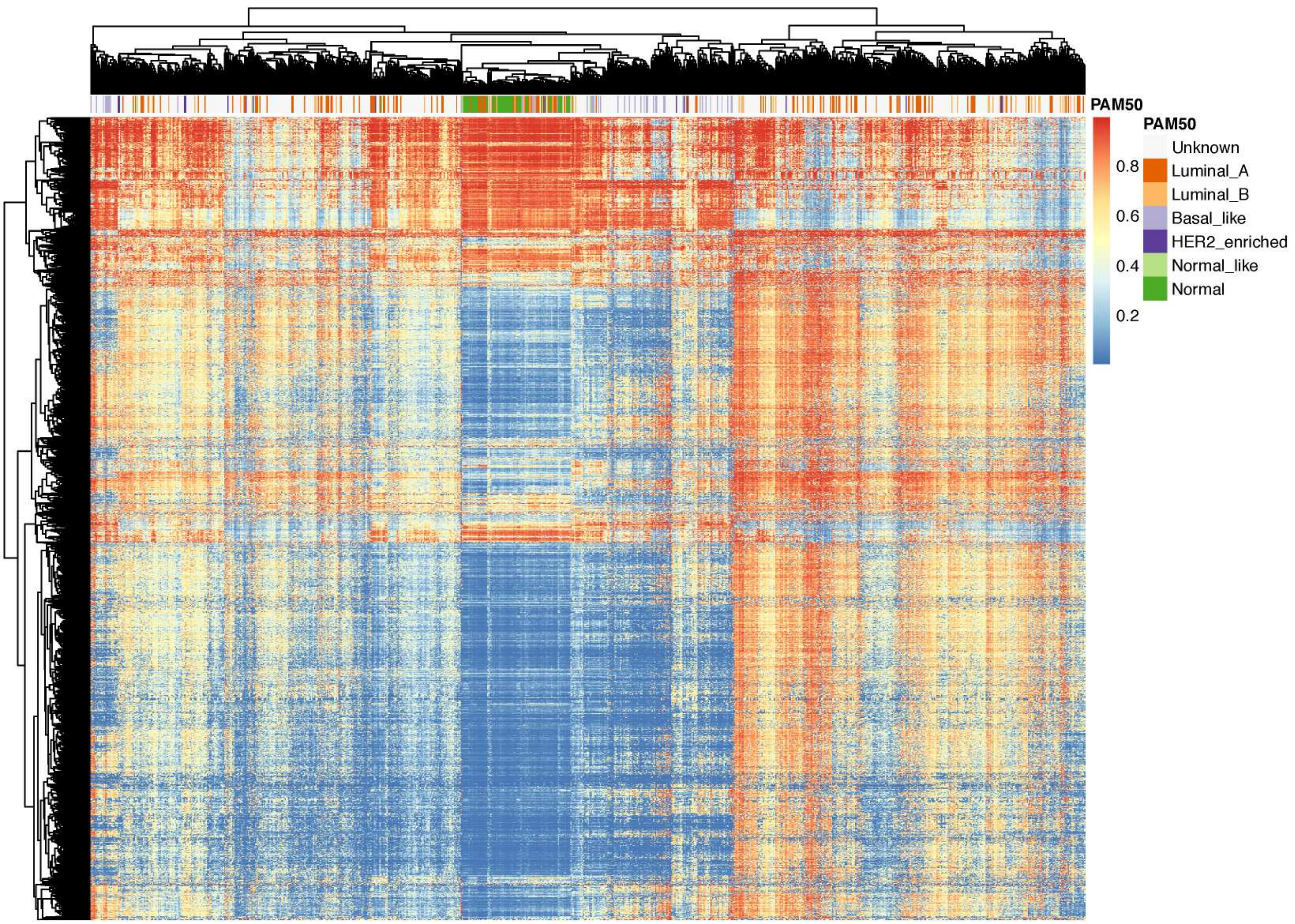

Figure S1. Unsupervised clustering of top 1% most variable probes of all the 870 TCGA-BRCA 450K samples.

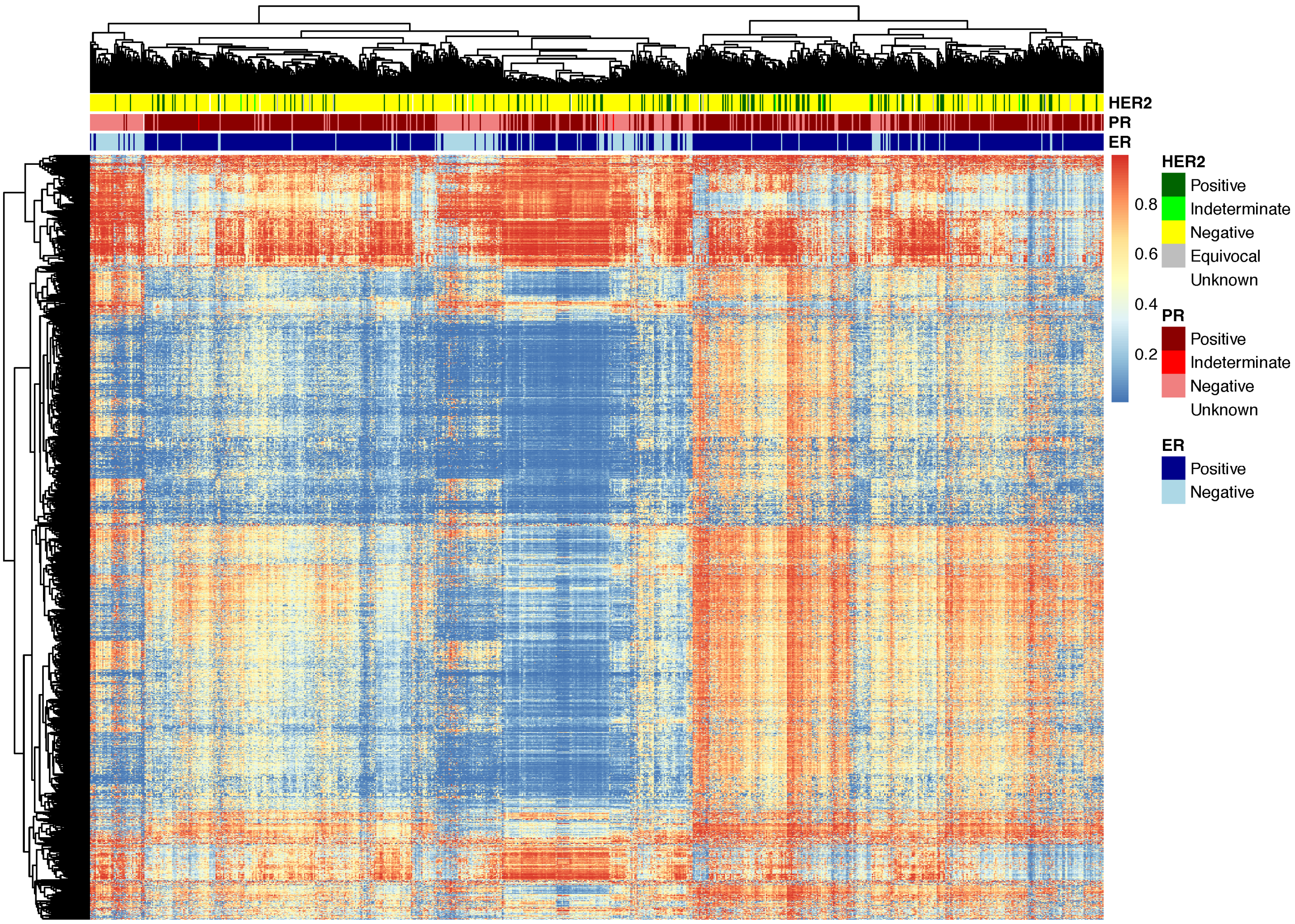

Figure S2. Unsupervised clustering of top 1% most variable probes of all the 870 TCGA-BRCA 450K samples with PR/ER/HER2 annotations.

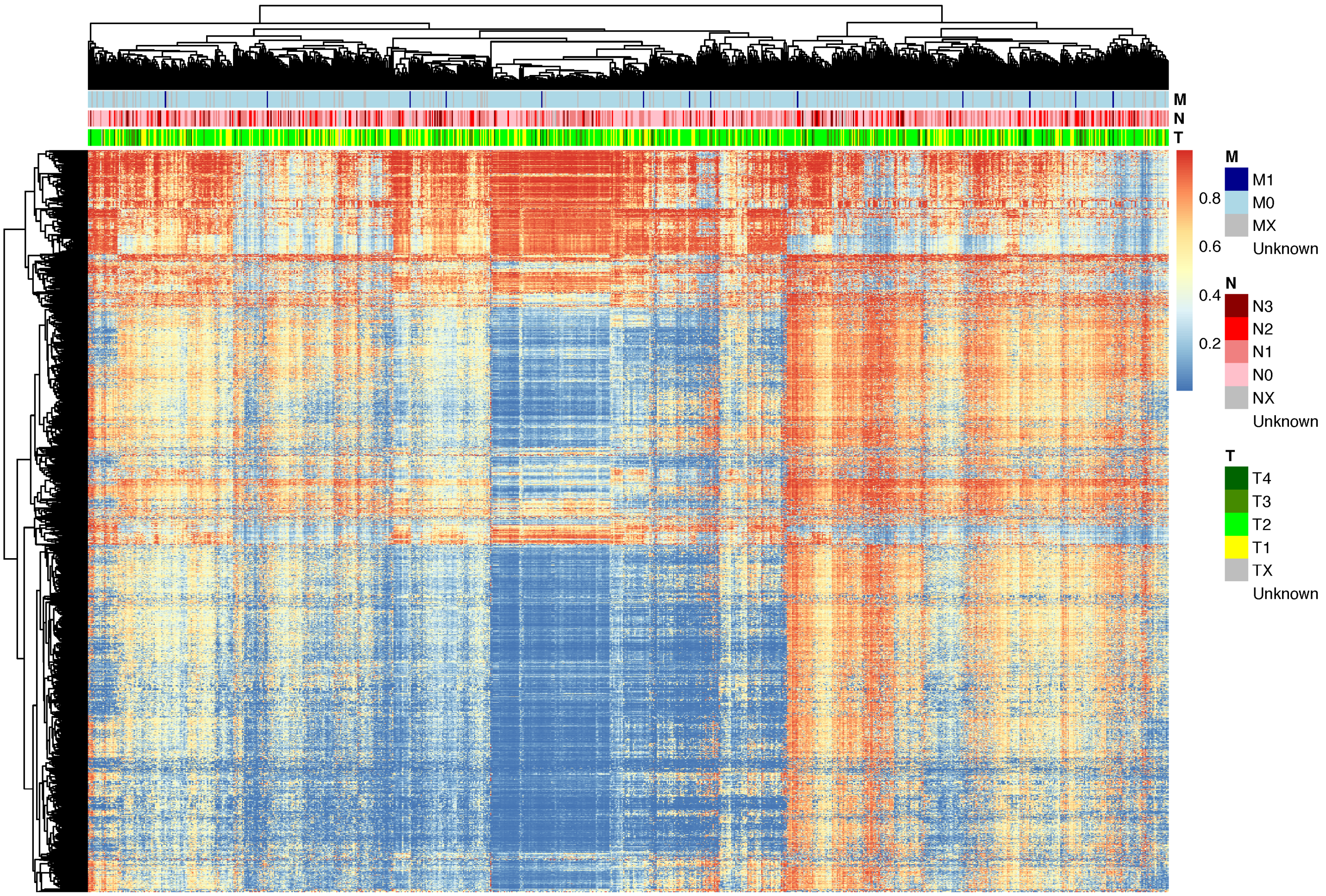

Figure S3. Unsupervised clustering of top 1% most variable probes of all the 870 TCGA-BRCA 450K samples with TNM annotations.

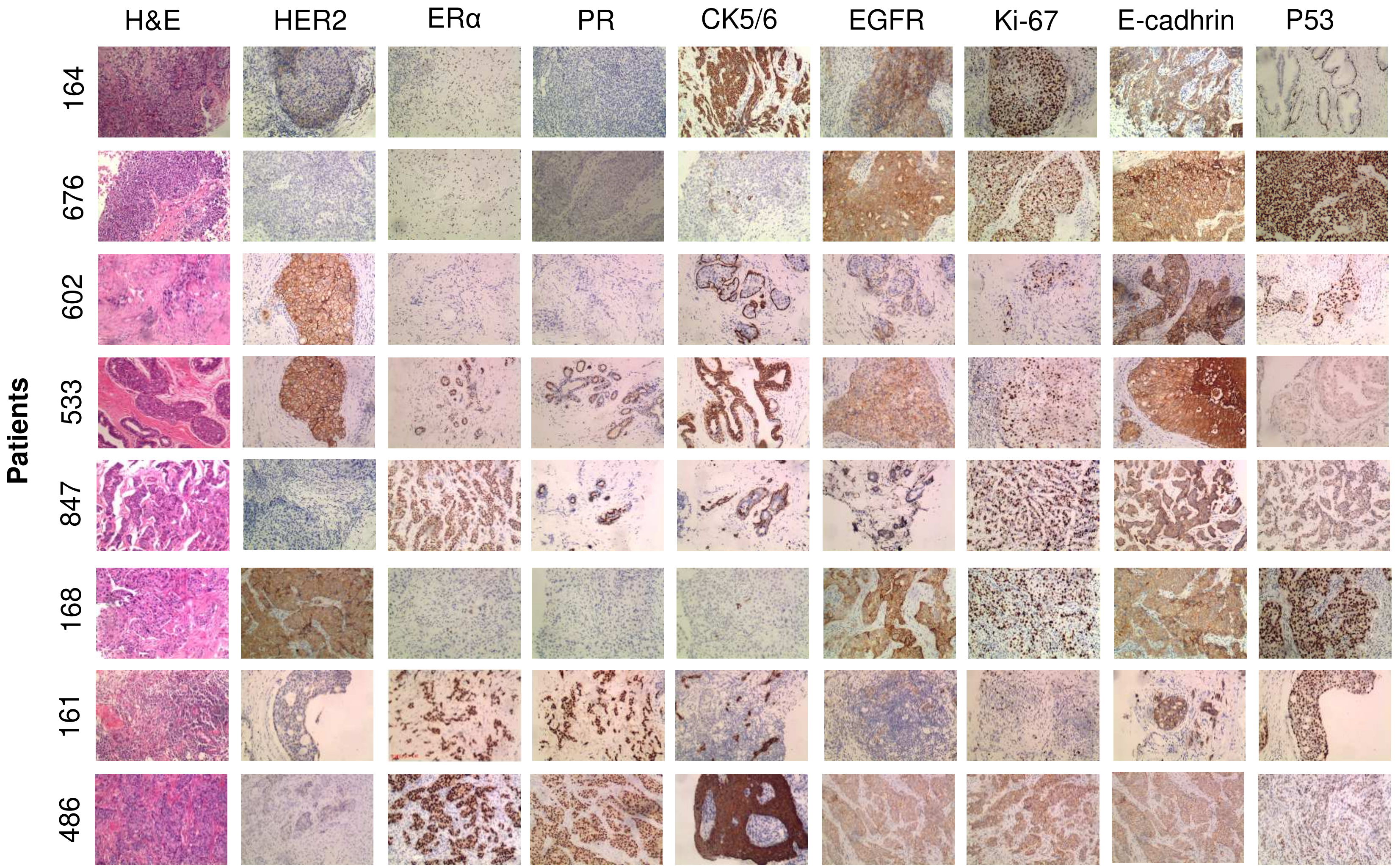

Figure S4. Histopathological analysis of selected breast cancer patients.

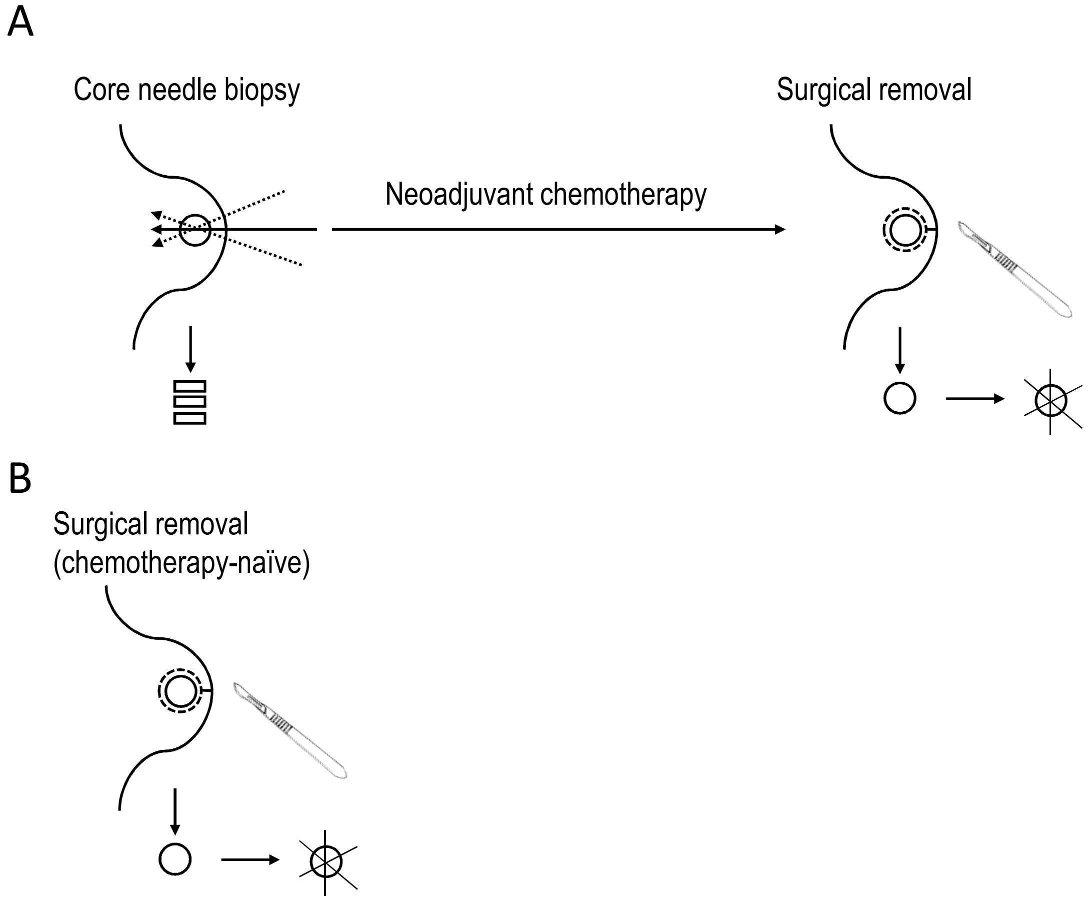

Figure S5. Sampling procedures. (A) Breast cancer patients were selected who underwent core needle biopsy sampling, followed by CET neoadjuvant chemotherapy regimens, and finally surgical removal of the breast tumors. Each patient derived 3-4 core needle biopsy specimens prior to NAC, and each post-NAC tumor tissue was spatially dissected into 6-7 sectors; **(B)** Breast cancer patients were selected whose breast tumors were surgically removed without neoadjuvant chemotherapy treatment. Each tumor tissue was spatially dissected into 6 sectors.

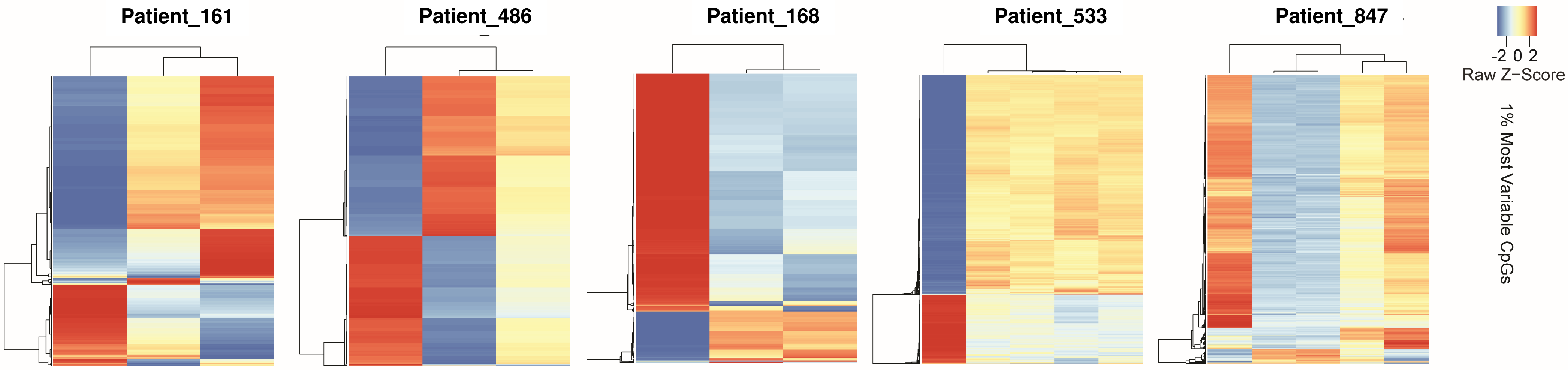

Figure S6. Unsupervised clustering of top 1% most variable probes of samples from 5 patients without chemotherapy separately.

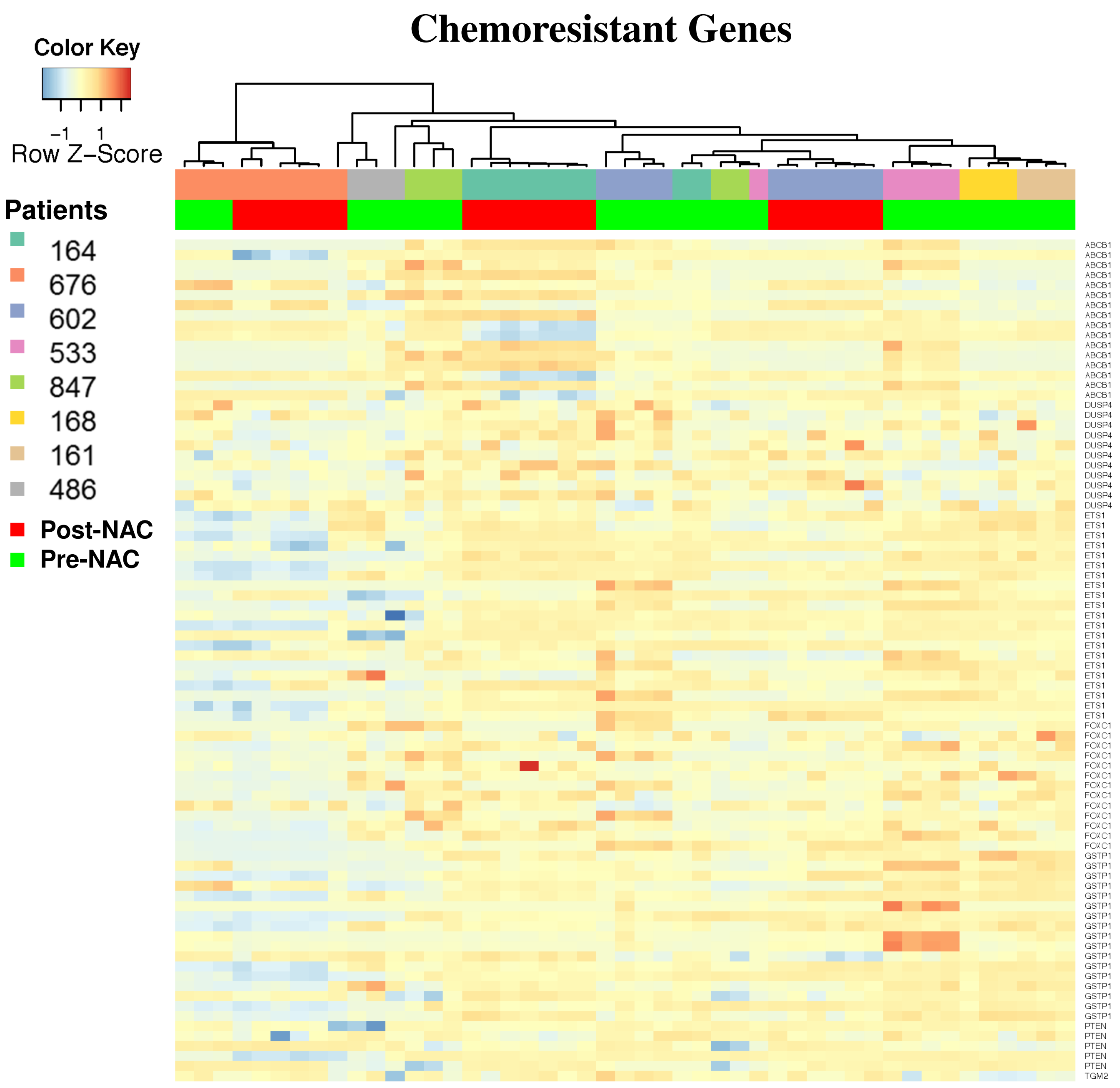

Figure S7. Unsupervised clustering of 450K array probes mapped to known genes associated to chemotherapy resistant: ABCB1, DUSP4, ETS1, FOXC1, GSTP1, PTEN and TGM2.

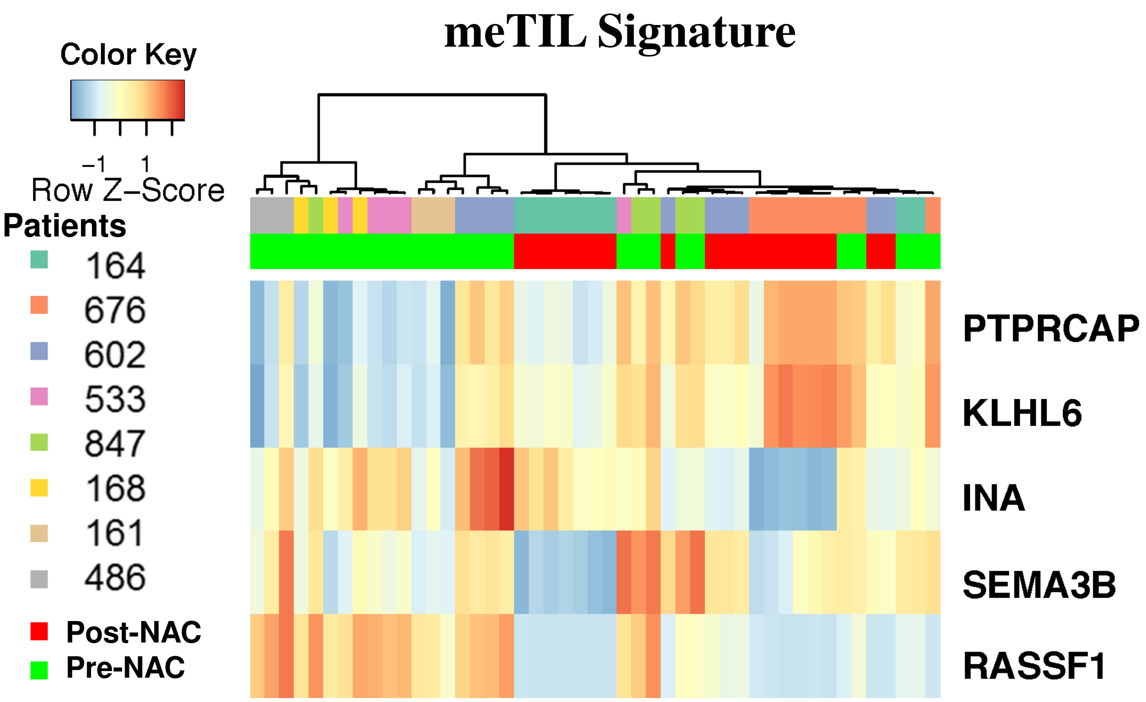

Figure S8. Unsupervised clustering of MeTIL signature probes of all the samples.

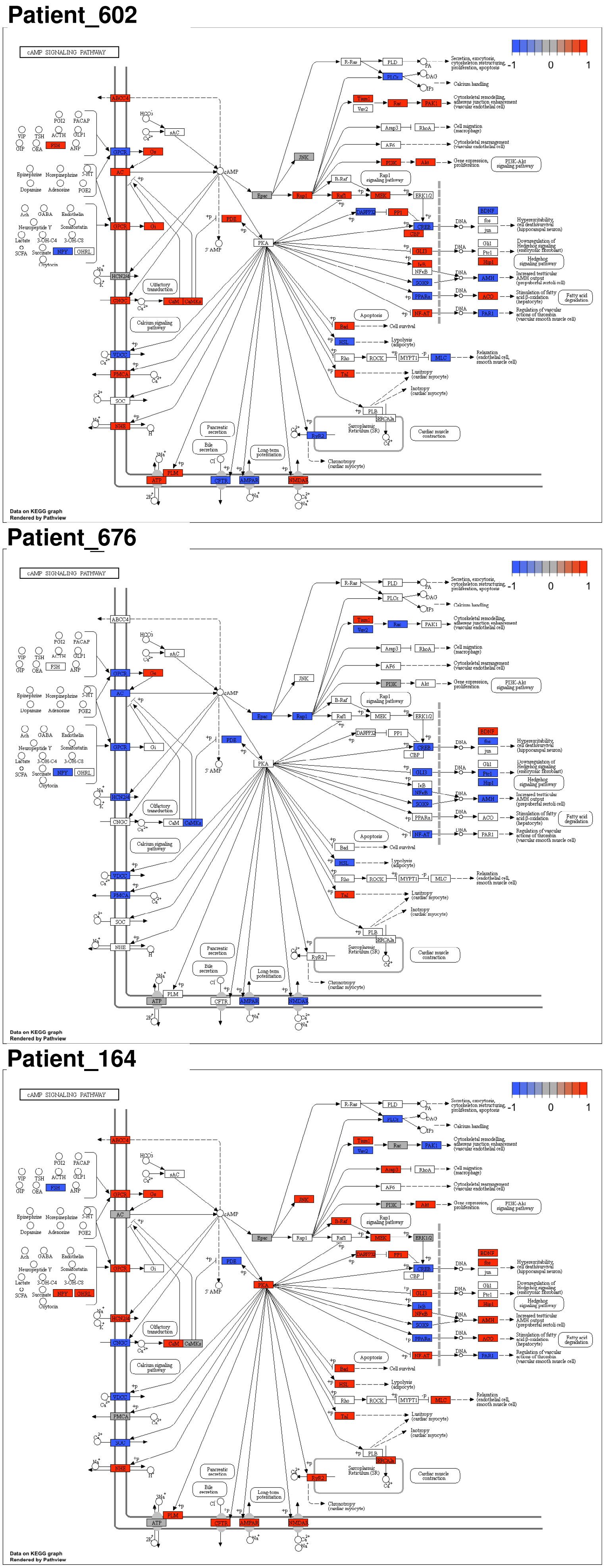

Figure S9. DM genes in KEGG cAMP signaling pathway in the 3 NAC-treated patients. Red color indicates hypermethylated genes; blue color indicates hypomethylated genes.

Patient_602

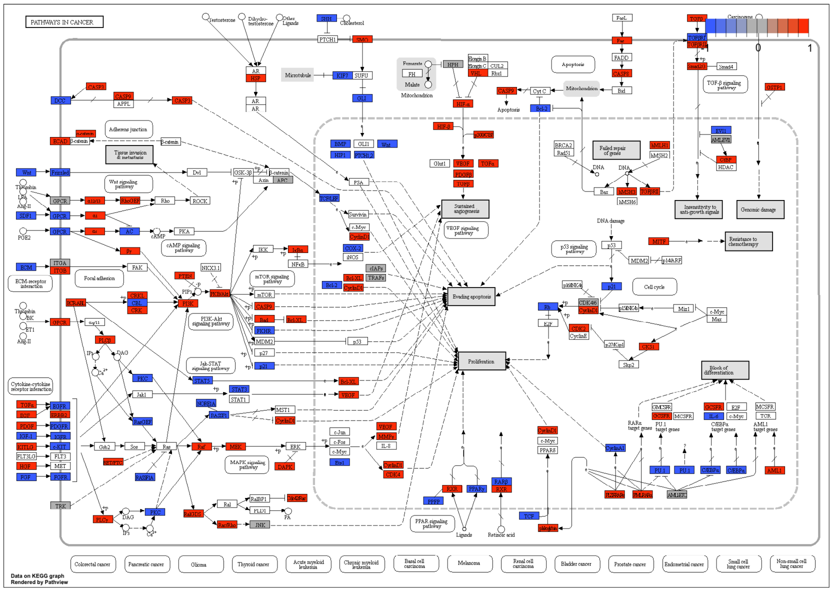

Patient_676

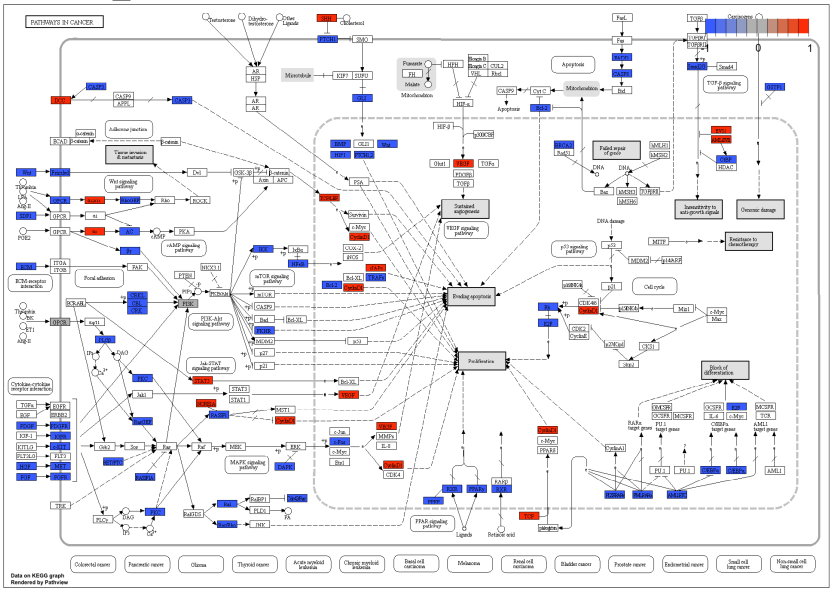

Patient_164

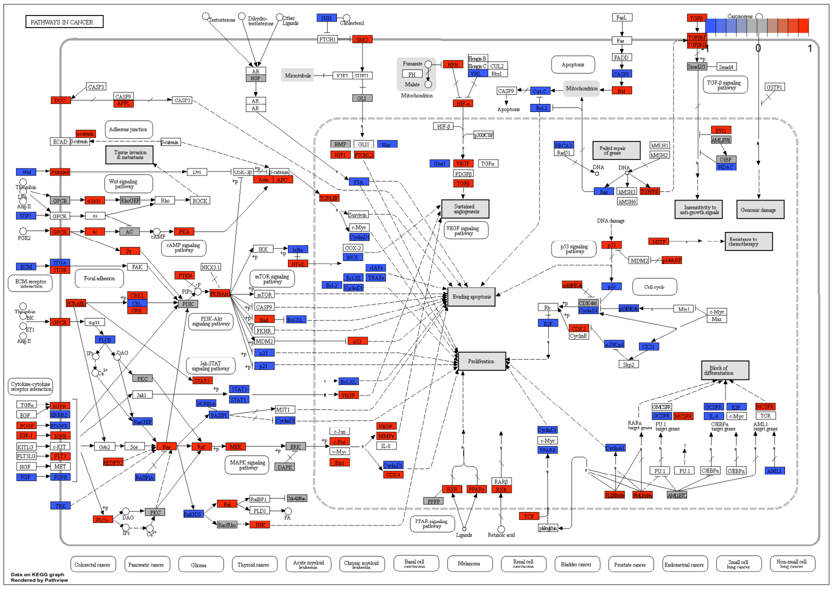

Figure S10. DM genes in KEGG Pathways in Cancer in the 3 NAC-treated patients. Red color indicates hypermethylated genes; blue color indicates hypomethylated genes.

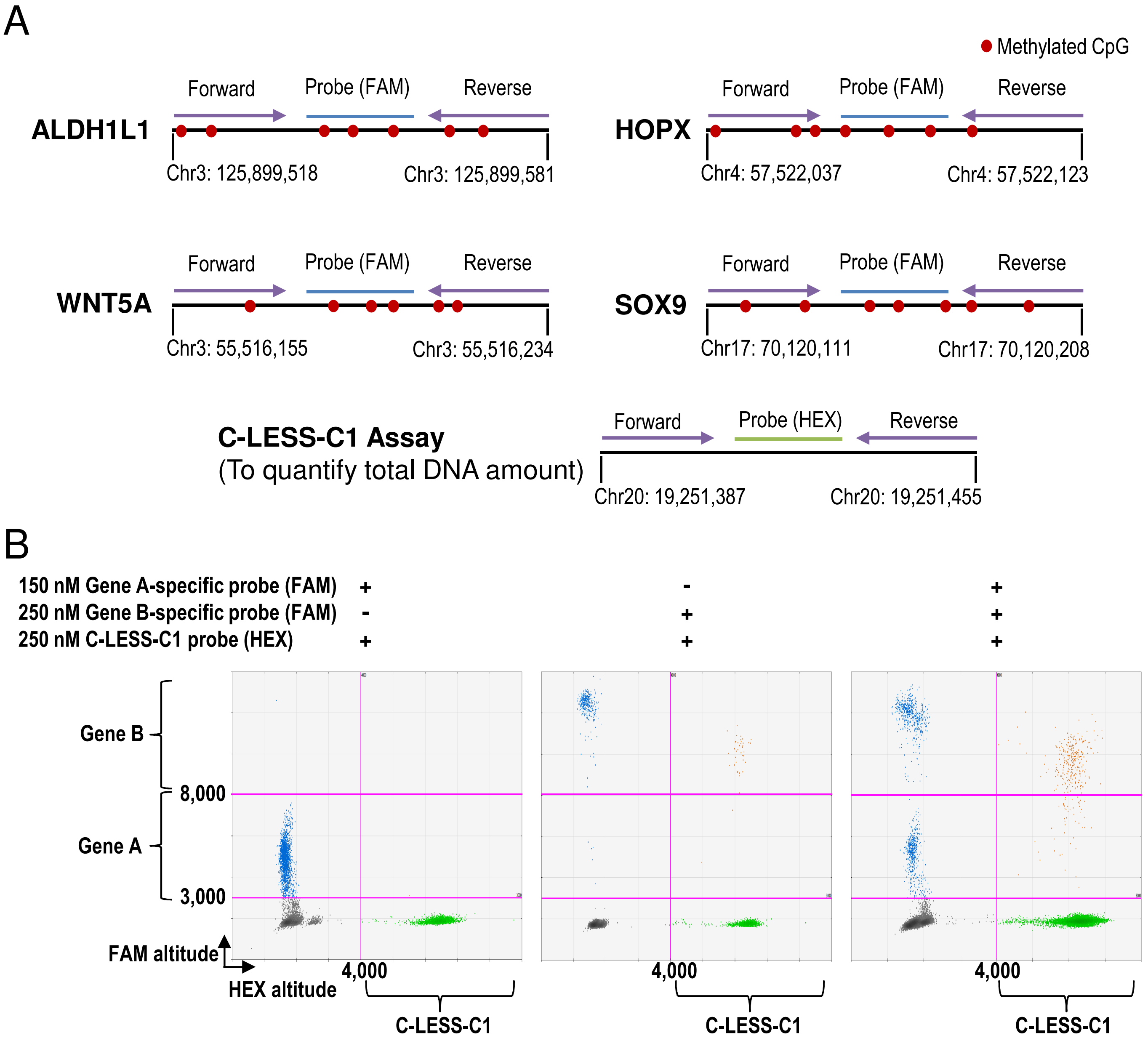

Figure S11. Establishment of multiplexed Methylight ddPCR. (A) Locations of CpGs in the MethyLight primers and probes and the amplicons for methylated loci of interest, and the C-LESS-C1 assay that amplifies a DNA strand without any cytosine to determine the total DNA amounts in each sample. Genomic coordinate is referred to UCSC hg19; **(B)** The C-LESS-C1 assay is measured by the HEX-labelled probe, meanwhile 2 genes of interest are measured by the 2 gene-specific FAM-labelled probes adjusted at different concentrations. The accuracy of ddPCR is sufficient to display the 2 gene-specific FAM-positive droplets at 2 distinctively separated FAM altitudes, enabling quantification of the 2 genes with 1 fluorescent channel. For the first assay, Gene A = ALDH1L1, Gene B = SOX9; for the second assay, Gene A = HOPX, Gene B = WNT5A. Thus, the 4 genes can be measured with only 2 assays.
